# LMAP: Lightweight Multigene Analyses in PAML

**DOI:** 10.1186/s12859-016-1204-5

**Published:** 2016-09-06

**Authors:** Emanuel Maldonado, Daniela Almeida, Tibisay Escalona, Imran Khan, Vitor Vasconcelos, Agostinho Antunes

**Affiliations:** 1 CIIMAR/CIMAR – Interdisciplinary Centre of Marine and Environmental Research, University of Porto, Terminal de Cruzeiros do Porto de Leixões, Avenida General Norton de Matos, s/n, 4450-208 Matosinhos, Portugal; 2 Department of Biology, Faculty of Sciences, University of Porto, Rua do Campo Alegre, 4169-007 Porto, Portugal

**Keywords:** Adaptive evolution, Software package, *PAML*, *codeml*, Codon substitution models, Multigene, Multi-core

## Abstract

**Background:**

Uncovering how phenotypic diversity arises and is maintained in nature has long been a major interest of evolutionary biologists. Recent advances in genome sequencing technologies have remarkably increased the efficiency to pinpoint genes involved in the adaptive evolution of phenotypes. Reliability of such findings is most often examined with statistical and computational methods using Maximum Likelihood codon-based models (i.e., site, branch, branch-site and clade models), such as those available in *codeml* from the Phylogenetic Analysis by Maximum Likelihood (PAML) package. While these models represent a well-defined workflow for documenting adaptive evolution, in practice they can be challenging for researchers having a vast amount of data, as multiple types of relevant codon-based datasets are generated, making the overall process hard and tedious to handle, error-prone and time-consuming.

**Results:**

We introduce LMAP (Lightweight Multigene Analyses in PAML), a user-friendly command-line and interactive package, designed to handle the *codeml* workflow, namely: directory organization, execution, results gathering and organization for Likelihood Ratio Test estimations with minimal manual user intervention. LMAP was developed for the workstation multi-core environment and provides a unique advantage for processing one, or more, if not all *codeml* codon-based models for multiple datasets at a time. Our software, proved efficiency throughout the *codeml* workflow, including, but not limited, to simultaneously handling more than 20 datasets.

**Conclusions:**

We have developed a simple and versatile LMAP package, with outstanding performance, enabling researchers to analyze multiple different codon-based datasets in a high-throughput fashion. At minimum, two file types are required within a single input directory: one for the multiple sequence alignment and another for the phylogenetic tree. To our knowledge, no other software combines all *codeml* codon substitution models of adaptive evolution. LMAP has been developed as an open-source package, allowing its integration into more complex open-source bioinformatics pipelines. LMAP package is released under GPLv3 license and is freely available at http://lmapaml.sourceforge.net/.

**Electronic supplementary material:**

The online version of this article (doi:10.1186/s12859-016-1204-5) contains supplementary material, which is available to authorized users.

## Background

Selection of beneficial mutations may cause the fixation of alleles conferring fitness advantage to the organisms of a population, which ultimately may result in the adaptive evolutionary diversification of life forms. Uncovering at the molecular level how this biological process of phenotypic diversity arises and is maintained in nature has long been of interest to the evolutionary biologist. In this regard, the advent of new genome sequencing technologies has remarkably increased the efficiency of contemporary molecular research [1–3]. In particular, significant progress has been made towards the discovery of protein-coding genes that may underlie adaptive evolution of phenotypes. This has prompted an enormous collection of new genome sequence data requiring fast and efficient specialized bioinformatics software for assisting researchers in downstream analyses [2, 3].

Currently most available tests of adaptive evolution are based on Maximum Likelihood (ML) codon-based models that assess the nonsynonymous (*d*_N_) to synonymous (*d*_S_*)* substitution rate ratio (ω = *d*_N_/*d*_S_), where ω can be greater, equal or less than 1, indicating positive, neutral or negative selection, respectively [4]. Although a large number of applications integrating this framework and accounting for codon-based likelihood models of evolution are readily available, the *codeml* program from the Phylogenetic Analysis by Maximum Likelihood (PAML) package [5] is the most widely used in the literature, statistically robust and accurate in examining selective pressure [6–11]. Henceforth, *codeml* will only refer to codon substitution models.

The evaluation of selection signatures is processed in two stages. First, *codeml* executes different model approaches, each of which uses different assumptions about how ω varies across a multiple sequence alignment (MSA) and/or phylogeny: (*i*) site-specific models (SM) [12, 13], (*ii*) branch-specific models (BM) [14, 15], (*iii*) branch-site specific models (BSM) [13, 16, 17] and (*iv*) clade-specific models (CM) [8, 18]. Second, for all models, a Likelihood Ratio Test (LRT) [12, 19, 20] is used to examine the goodness-of-fit between two nested models and determine which fits the dataset better (for details please see Supplementary Data in [8]).

We present a brief summary of the models here addressed and the reader is encouraged to see the involved references for further information.

The SM are generally applied to detect the presence of positively selected sites in the MSA. It employs different site class specific models: (*i*) the alternative classes which includes model 3 (M3), 2 (M2a) and 8 (M8) and, (*ii*) the null classes which includes model 0 (M0), 1 (M1), 7 (M7) and 8a (M8a). Models are pairwise compared (M0 vs. M3, M1a vs. M2a, M7 vs. M8, M8a vs. M8 [12, 21, 22]) using LRT. Whenever LRTs are significant, sites under selection are identified by the Bayes Empirical Bayes (BEB) analysis [13], except for the M0 vs. M3 comparison, since it does not allow detection of positive selection [16] and M3 does not provide the BEB estimation.

The branch related models (BRM—BM, BSM and CM) require an *a priori* partition of the phylogeny by implementing a branch labeling scheme allowing one to examine one or more lineages or even entire clades (e.g., [23–30]), usually defined as “foreground” and “background” branches or lineages [16]. Additional information on technical aspects can be found in PAML documentation.

The BM determines signals of divergence among lineages by examining whether changes in ω ratios vary significantly or not between branches [14–16]. Although various model comparisons are possible, this generally involves performing two LRT comparisons among three models [14, 28]. The first is accomplished by testing the null M0 against an alternative with a two-ratio unconstrained (TrU) model (M0 vs. TrU). If TrU fits the data better, then the second LRT comparison can be tested in order to validate signals of divergence. Here, TrU is tested against a two-ratio constrained (TrC, where ω = 1) model (TrC vs. TrU). Because the BEB analysis is not quantified in BM, positively selected sites cannot be inferred.

The BSM arose from the extension of the SM and BM and allows the detection of episodic selection occurring along few lineages [7, 16]. Here ω is allowed to vary both among sites and lineages, enabling inter-specific comparisons and detection of selection in a subset of sites within a subset of branches of the phylogenetic tree [16, 31]. In the phylogeny only two partitions are possible [32], (*i*) one configuring a model that allows positive selection on the foreground branches, the alternative model A (MA), and (*ii*) the other, a model that allows neutral and negative selection both on the foreground and background lineages, the null model A1 (MA1, where ω = 1) [13, 17]. In case of a significant LRT in this test (MA1 vs. MA), sites under positive selection can be inferred with high posterior probabilities through the BEB analysis.

Similarly, to the BSM, the CM can test for variation in selection pressures acting among sites and lineages, allowing the detection of divergent selection among clades, whether in the foreground or background branches. Under CM, a phylogeny can incorporate more than two partitions [8, 18]. Here the alternate model C (CmC) [18] estimates several separate ω ratios for two or more clades and is compared to a null model 2a_rel (M2a_rel), by applying a constraint enforcing ω to be fixed among clades [8]. The significance of site-specific divergence among clades is established via a LRT comparison between the two models (M2a_rel vs. CmC) [8, 32]. If the CmC is significant, then the BEB analysis can be used to identify sites experiencing divergence among clades. To further decide if divergently selected sites among clades are significantly under the action of positive selection, the value ω of the divergent site class is constrained to be equal to 1 and compared against an unconstrained CmC [11, 32].

SM, BM, BSM and CM LRT comparisons are respectively summarized in Additional file 1: Tables S1–S4 (bottom).

The *codeml* models constitute a well-defined workflow for analyzing genome-wide data and documenting selection in protein-coding genes. However, it can be highly challenging in practice due to the huge amount of information, as data integration and analysis involves often multiple tasks that need to be manually performed by the researcher, including gathering and organizing input data [33], manipulating software configuration files, and running and analyzing the results. Specifically in the *codeml* workflow, it is necessary to generate (*i*) MSAs, (*ii*) phylogenetic trees, (*iii*) edit the parameter files, (*iv*) organize files in folders, (*v*) run *codeml*, (*vi*) collect all necessary ML parameter estimates and (*vii*) estimate all LRT comparisons in spreadsheet documents. Moreover, the challenge is even greater when performing these tasks repetitively for multiple datasets (i.e., MSAs and phylogenetic trees), making the whole process very tedious, error-prone and time-consuming.

To overcome such difficulties several bioinformatics resources have been developed. They can be organized in two paradigms: single-task (one instance, one execution: *JCoDA* [34], *Armadillo* [35], *PAMLX* [36], *IMPACT_S* [37]) and multi-task (one instance, several executions: *IDEA* [38], *gcodeml* [39], *POTION* [40], VESPA [41]). In the single-task software group, SM executions are possible in all software, while BM and BSM are also possible in *Armadillo* and *PAMLX*. This last one additionally allows CM executions. Regarding the multi-task software group, SM executions are possible in *IDEA*, *POTION* and *VESPA*, while BSM are possible in both *gcodeml* and *VESPA. IDEA* further allows BM executions. In this group, *IDEA*, *gcodeml* and *VESPA* provide parallelized and/or distributed executions by including cluster or GRID functionality. Despite providing an important advancement in large scale analyses, they are however, too complex to install and configure [34], and usually require unavailable infra-structures or informatics skills. For instance, the *gcodeml* is mainly intended for production managers [39]. Such difficulties are minimized by the recent *POTION* software aimed at the more ubiquitous multi-core environment. Here a single workstation may currently offer 30 or more cores by combining two or more CPUs, thus providing a reasonable amount of processing capacity.

In addition to the desktop-based applications described, there are also web-server implementations available, namely *PSP* [42], *PhyleasProg* [43] and *Selecton* version 2.2 [44]. All involve SM, but *PSP* and *PhyleasProg* also include BSM analyses.

To our knowledge, from the available literature and from the mentioned multi-task software group, detection of positive selection is performed mainly using SM, while none of them considers the CM approach (see also Additional file 9 in [40]). Despite all these attempts, there is still the need of a software which simplifies the manual manipulation required for evolutionary analyses, while still including all the *codeml* models.

Here we propose LMAP (Lightweight Multigene Analyses in PAML), a high-throughput user-friendly software package designed to simplify evolutionary analyses performed with any of the described codon substitution models (SM, BM, BSM and CM). LMAP package is composed of six command-line and interactive Perl [45] applications designed to handle step-by-step the *codeml* workflow, thus minimizing user intervention. Although there are six applications, one of them (*lmap.pl*) further combines all others hereby reducing the *codeml* workflow to a single execution.

To enable LMAP trial and testing, an example dataset consisting of the mitochondrial DNA of 20 freshwater and terrestrial turtles is provided in the archive.

In the following sections, we present LMAP development, devised *codeml* templates, how input is simplified and how scheduling copes with workstation CPU capacity. Finally, we present the functioning of each LMAP application, discuss potential future developments and introduce the example dataset with which are performed benchmarking tests.

## Implementation

### LMAP development

LMAP package was implemented in Perl [45] and has been tested in Linux/UNIX and MacOS. It consists of six command-line and/or interactive applications, (*i*) *gmap.pl*, (*ii*) *cmap.pl*, (*iii*) *mmap.pl*, (*iv*) *imap.pl*, (*v*) *omap.pl* and (*vi*) *lmap.pl*. Additionally, four specific LMAP library modules (*MyUtil.pm*, *MyNotify.pm*, *MyPAMLInfo.pm* and *MyPhylo.pm*) support the execution of these applications.

LMAP requires the Comprehensive Perl Archive Network (CPAN) [46] modules in five cases: (*i*) in *gmap.pl*, for parsing and editing of Newick tree files (BioPerl [47] module); (*ii*) in *mmap.pl,* for email functionalities and interactive monitoring of *codeml* parallel executions (for which are required the UNIX *sendmail* [48] and *screen* [49] utility programs); (*iii*) in *gmap.pl* and *omap.pl*, for interactive modes; (*iv*) in *omap.pl*, for statistics functions involved in estimation of LRTs; and *(v)* in all applications, for handling files and directories.

Although BioPerl modules enable PAML results processing, its implementation is limited to users with programming skills. By contrast, our package implements all necessary functions, excluding the cases mentioned above hereby requiring minimal installation efforts. Such necessary functions include specific procedures in the *imap.pl* application to allow the retrieval of ML parameter estimates.

To alleviate the installation of CPAN modules and utility programs we have included the *install.pl* application (see also the Availability and requirements section).

### LMAP management of *codeml* parameters and templates

Since all codon substitution models (SM, BM, BSM and CM) require different *codeml* control file configurations, we have defined nine templates (Additional file 1: Tables S1–S4). Two templates are used for SM (M0/1/2/3/7/8 and M8a) (Additional file 1: Table S1), three for BM (M0, TrC and TrU) (Additional file 1: Table S2), two for BSM (MA and MA1) (Additional file 1: Table S3), and two for CM (CmC and M2a_rel) (Additional file 1: Table S4). Some parameters on these templates are automatically adjusted by our software, such as input dataset (*seqfile* and *treefile*), translation table code (*icode*), *NSsites*, *kappa (k)* and *omega* (*ω*) values. Before getting started, the user is encouraged to verify the remaining parameters for each template and make any necessary adjustments, which will remain applicable until new modifications are enforced. In order to detect and avoid local optima [50], several values of *k* and *ω* parameters are by default defined in *gmap.pl* (Additional file 1: Tables S1–S4). To this end, any selection of values, can be used to perform independent executions for the same dataset.

### LMAP choice of selection models and input files

Here we describe how LMAP simplifies input by asking the researcher to specify the selection models in the dataset, ensuring the correct associations of MSA and tree files in the templates.

This is accomplished when naming the MSA file(s) and the phylogenetic tree file(s). In the case of MSA file(s), three key elements are necessary: (*i*) any identity or abbreviation of the protein-coding gene(s), (*ii*) model(s) identity(ies) to be applied, which could be run one or more at a time (‘s’, ‘b’, ‘w’, ‘c’ letters representing SM, BM, BSM and CM, respectively) and (*iii*) the appropriate *icode* parameter value. Thus, the MSA identity is represented as [GeneName]_[sbwc][icode].fasta (without brackets). In the case of the phylogenetic tree(s), the nomenclature depends on the existence of labeling. Tree labeling is necessary when examining BRM, but not with SM. Therefore, these two procedures require different identities. In the SM case, the user needs to type the same gene name as its correspondent MSA file and the SM letter ‘s’, resulting in the format [GeneName]_s.nwk (without brackets). In the BRM case, tree labeling depends on the branch partitions scheme (hypothesis) defined by the researcher. Hence, the tree file should be named after the hypothesis reference (HR) and include one or a combination of BRM (letters ‘w’, ‘b’, ‘c’), which is represented as [HR]_[wbc].nwk (without brackets). It is worth noting that in the SM case, the MSA will only be combined with a similarly named phylogenetic tree, that is, [GeneName]_[sbwc][icode].fasta with [GeneName]_s.nwk (without brackets) (e.g.: MSA ATP6_sbwc1.fas with tree ATP6_s.nwk).

An advantage of this design is that it allows the user to combine in a single step one or more, if not all unique MSAs, with as many as required phylogenetic tree files (or hypotheses) to be run, regardless of the models specified (letters ‘s’, ‘w’, ‘b’, ‘c’) (e.g.: ATP6_sbwc1.fas with TWC_w.nwk and with 2WA_b.nwk). To conclude, in order to combine an MSA and a tree, the same model letter must be specified in both input files names. Please see the manual included in LMAP package for more information.

### LMAP scheduling of *Codeml* executions

In this section, we describe how the *mmap.pl* application was designed to cope with several *codeml* executions and its relation with the workstation CPU capacity.

At this stage, the input files together with *codeml* control files are ready for execution in subfolders within a base folder, which we refer to henceforth as directory structure.

The *mmap.pl* allows the user to run as many *codeml* tasks as desired. Because the total number of tasks can be very large, most probably surpassing the total number of CPU cores, the application provides the command-line option (CLO) -n to define the maximum tasks to be run. This will define the maximum number of cores utilized (one task per core). When used, a value for this option must be defined, or otherwise the value is automatically estimated. In this case, the application quantifies an approximate number of available CPU cores, which in consequence defines the maximum number of *codeml* tasks to be run. This is achieved by calculating the difference of total number of cores to the overall CPU load. Under these circumstances, the quantification of available CPUs by the application makes sense, since it maximizes the performance of the whole scheduling.

It is noteworthy, that the greater the number of CPU cores available, the faster the execution of the *mmap.pl* application will be. Nevertheless, this is highly dependent on the user’s workstation configuration (CPU, memory, etc.). Please see the Example dataset and benchmarking section for more information.

## Results and discussion

### LMAP applications and functionalities

**Fig. 1:**
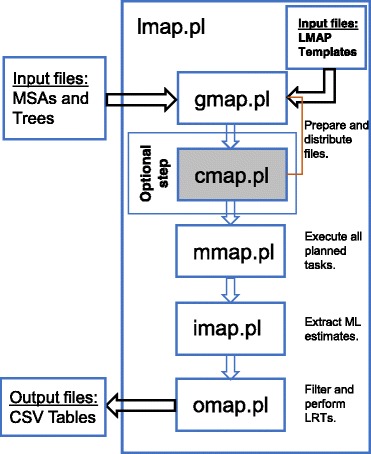
LMAP workflow. Flowchart exhibiting the *lmap.pl* workflow where five LMAP applications are sequentially combined to gather results from each *codeml* execution prepared and executed in the directory structure

The LMAP software package consists of six applications. The first five are independently applied to accomplish one-by-one the system workflow, which should be accomplished in the following order: *gmap.pl, cmap.pl, mmap.pl, imap.pl, omap.pl*. The sixth and last application, *lmap.pl,* automatically combines all others and facilitates the workflow in one step (Fig. 1). We describe next the functionality of each application in an orderly fashion and according to several related command-line options.

**Fig. 2:**
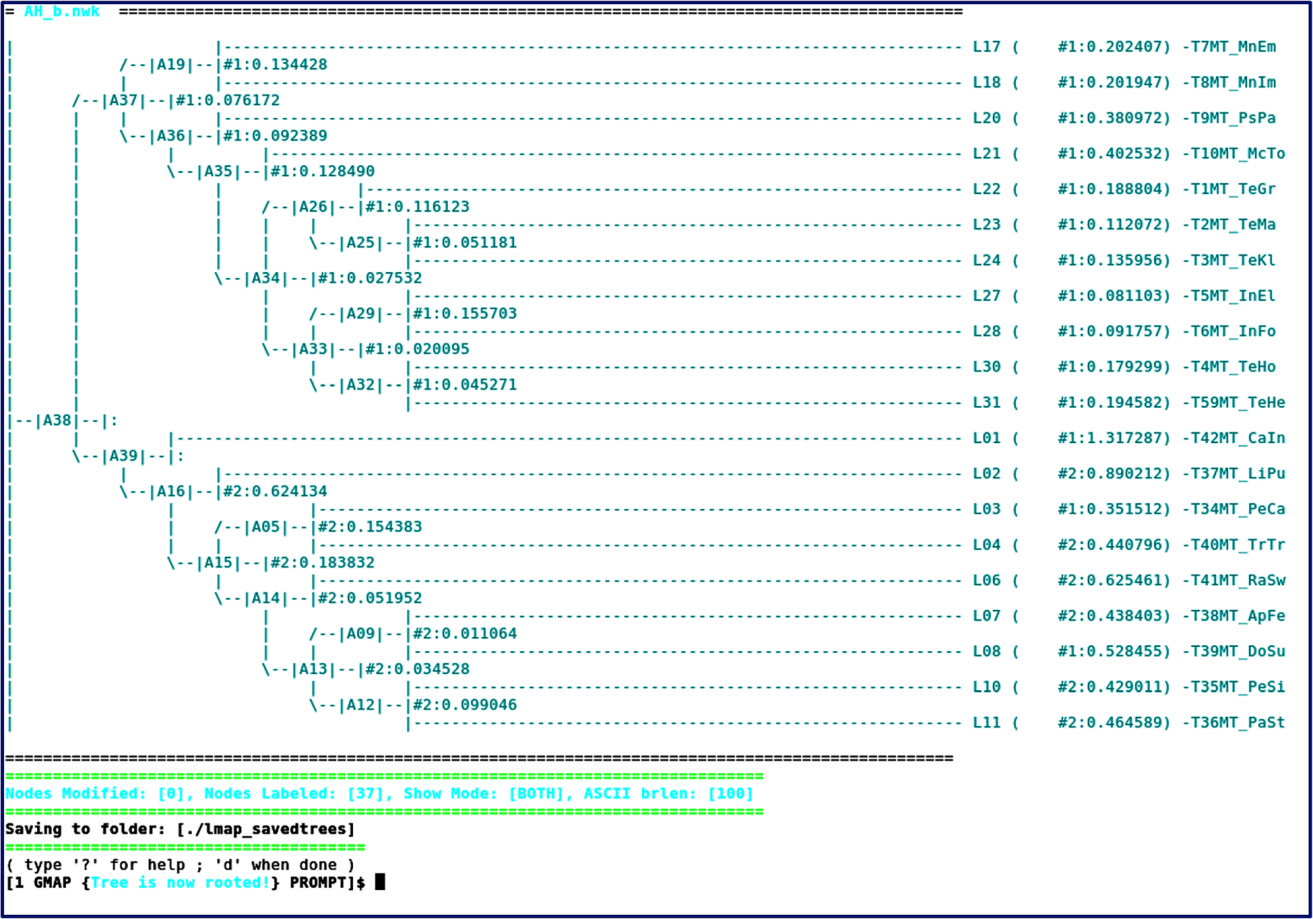
Tree editing interactive screen of gmap.pl. The phylogenetic tree file (from the included dataset) is displayed as a cladogram, allowing the user to make the necessary labeling. This screen shows various information (from left to right), such as the total number of nodes modified or affected, the total number of nodes labeled, the current selected display mode, which enables alternative display of phylogenetic tree information (i.e., bootstraps, branch lengths, both or none) and the cladogram branch length. The interactive commands for labeling and other operations can be consulted through an interactive help menu, by entering the “?” character in this screen or through the command-line option “--help” (as in *gmap.pl --help*), which will print the help into a specific text file in current directory (see Additional file 2: Figures S1 and S2)

The *gmap.pl* provides two functions: *(i)* generation of the directory structure and *(ii)* editing and/or labeling of phylogenetic trees. In the first, *codeml* input files are organized based on input datasets, CLOs and user definitions (Additional file 2: Figure S1). The option -m, enables the selection of which *codeml* models to run (letters ‘s’, ‘w’, ‘b’, ‘c’), hereby selecting the input files which have the same indication (see Implementation–LMAP choice of selection models and input files). Moreover, the options -K and -O aid in specifying *k* and/or *ω* values for the same dataset to avoid local optima [50], resulting in multiple executions starting from different initial parameter values. The second *gmap.pl* function is accessed by specifying the CLO -t, instead of -T and enables an interactive mode (Fig. 2) during which a cladogram character-based layout is displayed with numbers identifying tree nodes. Based on the researcher’s *a priori* hypotheses, specific branches (PAML label #N) or clades (PAML label $N) of interest may be labeled into foreground or background (Additional file 2: Figure S2), where N is the branch partition number (see PAML documentation for tree labeling and references therein).

The *cmap.pl* (Additional file 2: Figure S3) is designed to allow users to make additional changes to any parameters of the *codeml* control files available in the directory structure. These modifications do not affect the LMAP templates and any adjustment to the parameters can take place at any time, before the *codeml* executions.

**Fig. 3:**
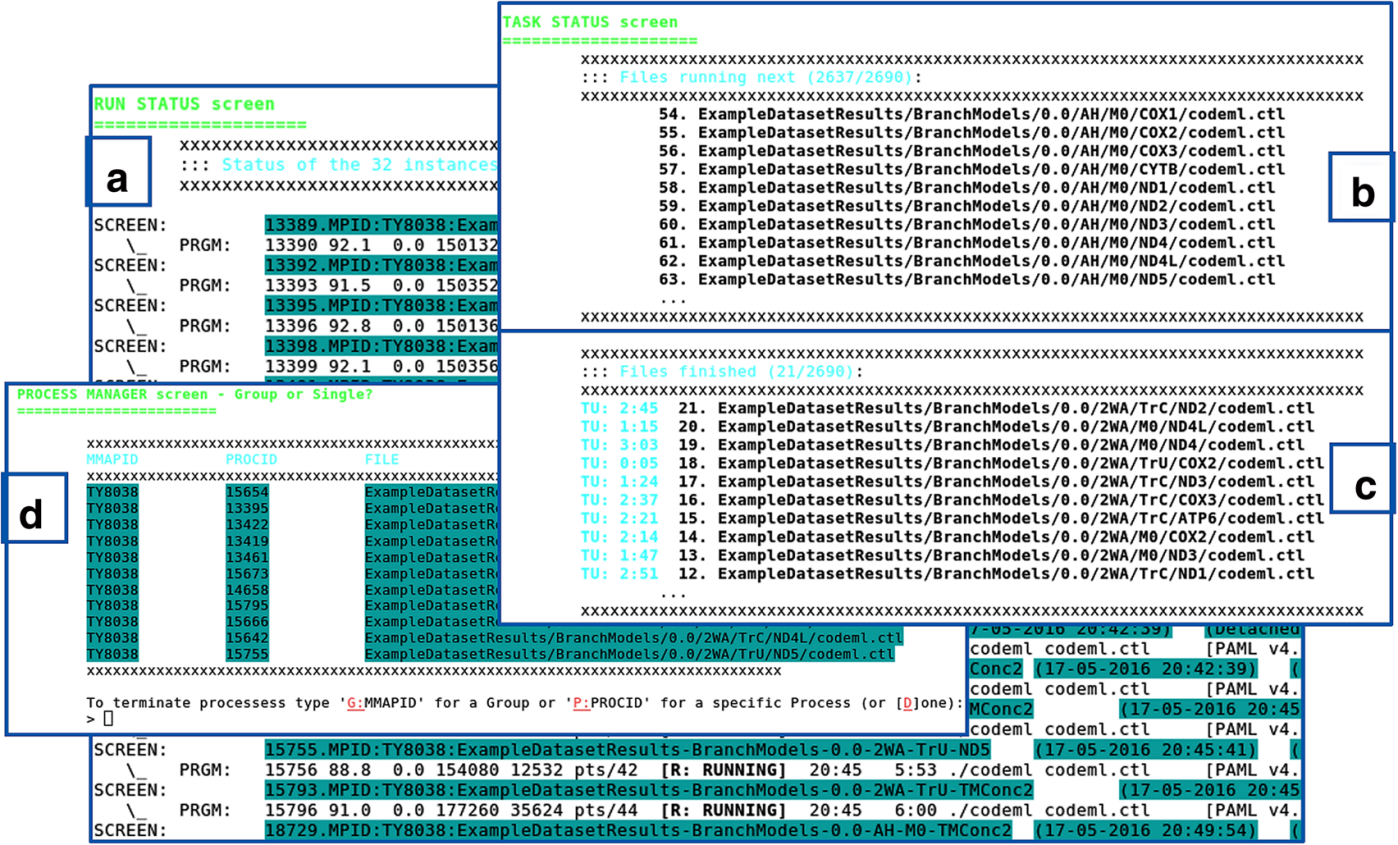
Interactive functioning of mmap.pl. **a** default or main “Run Status” screen presenting the currently running *codeml* tasks; by pressing “2”, shows the “Task Status” screen, showing **b** the tasks that will be running next (first ten) and **c** the tasks currently finished (last ten) (press "1" to go back to "Run Status" screen); **d** when interrupting the execution of *mmap.pl* (by typing “Ctrl-c” or “Ctrl-\”), beyond the choice of quitting, the user has also the choice to proceed to the built-in process manager here presented, allowing the termination of specific tasks. In this case, it is possible to terminate a group of tasks by typing “G:MMAPID” or a single task “P:PROCID”. The identifiers for MMAPID and PROCID are shown in the table, in the respective columns

The *mmap.pl* (Fig. 3 and Additional file 2: Figure S4) application aims to run the *codeml* program on the directory structure. During this phase, the user is able to monitor the *codeml* instances that are currently in execution (in screen 1) (Fig. 3a), those which will be executed (Fig. 3b) and those that have finished (Fig. 3c) (both in screen 2). Through the monitoring, the user is able to quickly understand whether the *codeml* instances are running correctly (Fig. 3a – “[R: RUNNING]” tag), or otherwise are hanging or waiting for the user reply, which could mean invalid dataset specifications. Having found unwanted or problematic instances, these can be terminated by accessing the built-in process manager screen (Fig. 3d). Another useful functionality of *mmap.pl*, is that it provides a non-mandatory email notification, which occurs as soon as the batch of instances is completed.

After *mmap.pl* terminates, the resulting information from all the analyzed models can be extracted using *imap.pl* (see Additional file 2: Figure S5). This information is organized in a CSV file and will contain each model organized by rows, while its ML parameters estimates (omegas, log-likelihoods, kappas, proportions, posterior probabilities, among others) are organized by columns.

**Fig. 4:**
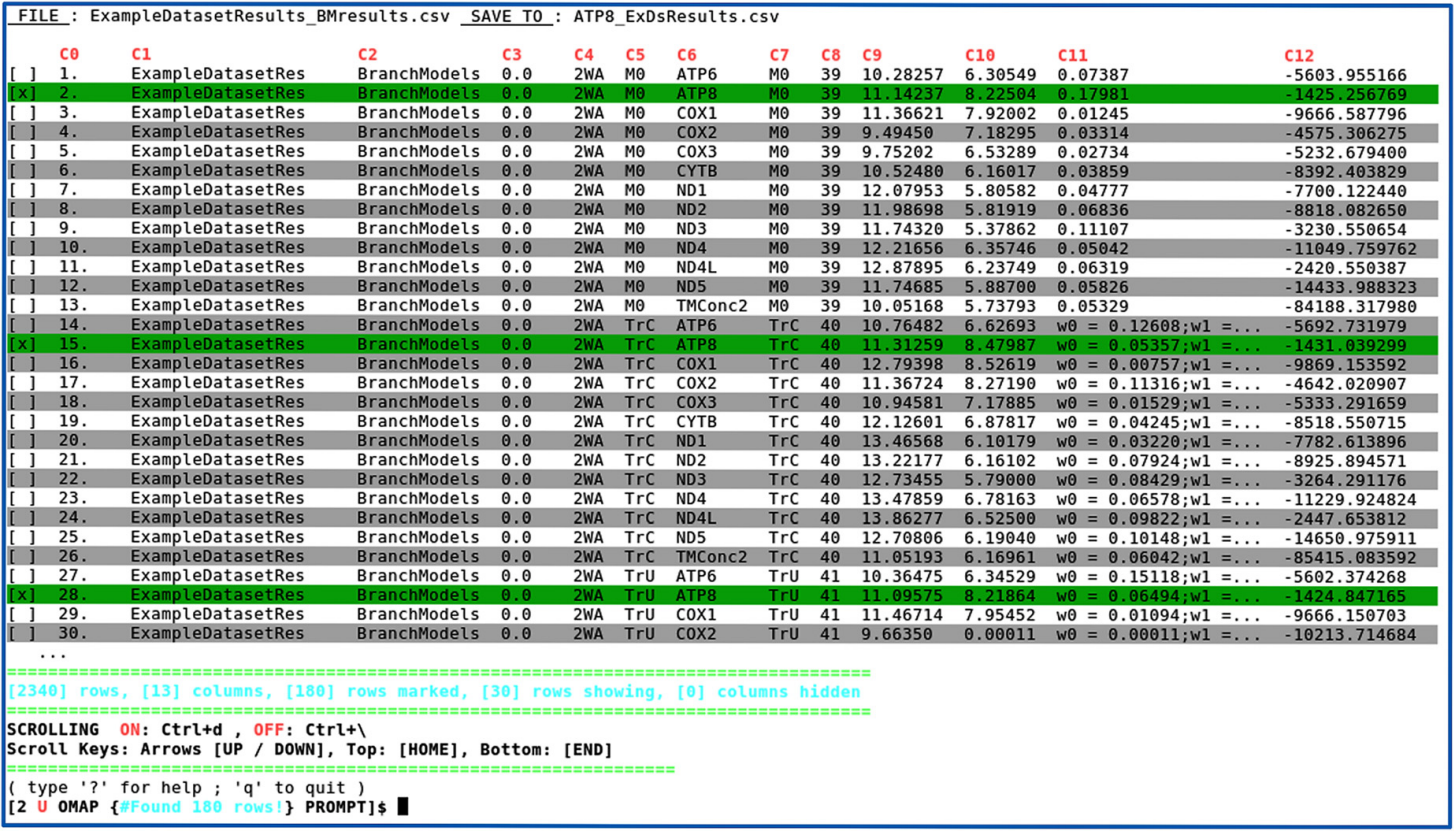
Interactive functioning of omap.pl - User Table. The resulting CSV table containing the BM data from *imap.pl*, where each row contains the absolute path to the corresponding *codeml* results file in the directory structure. This path is decomposed in columns by *omap.pl*, whereby each subfolder name constitutes a column. This additional on-screen information complements the *codeml* maximum likelihood parameter estimates simplifying overall data perception, advantageous for organization processes. At the top, for simpler use across the interactive commands, the column names (*in red*) are shown as “C + number” (see also Fig. 5), whereas the original CSV column names, are revealed, through the command “fh” (Additional file 2: Figure S7). It is possible to adjust visible table information, by scrolling and by (un)hiding columns or defining number of visible rows. Below the table, from left to right, various information is shown (*in cyan*), such as the total number of rows and of columns, the number of selected rows, the number of visible rows and the number of hidden columns. Scrolling information is shown below; activate scroll by typing “Ctrl-d”, use arrow keys to scroll up or down, “Home” key to go to top or “End” key to go to bottom. When finished, deactivate scroll by typing “Ctrl-\”, to enable interactive commands processing. Below, the table currently shown (“User Table”) is indicated by the letter “U” (*in red*) in the OMAP PROMPT line. The interactive commands are consulted in two ways, either through a help screen, triggered by entering the “?” command, or through the CLO “--help” (as in o*map.pl --help*), which will print the help into a text file in the current directory (see Additional file 2: Figures S6 and S7)

**Fig. 5:**
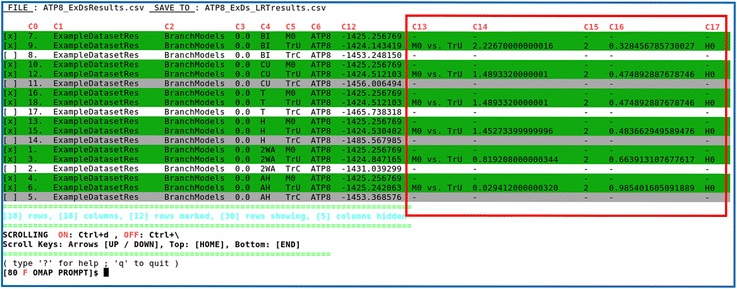
Interactive functioning of omap.pl - Final Table. The data shown consists of all ATP8 hypotheses with LRT estimations. All hypotheses were separated and organized from the initial User Table (Fig. 4) using *omap.pl*. The five LRT columns delimited by the red square, were appended after entering the “plrt 0.05” command (Additional file 2: Figure S7). These columns are always defined in the following order, (*i*) the LRT comparison (column C13), whose parameter estimates define the following columns; (*ii*) deltaLnL (column C14), for twice the difference on the lnL scores; (*iii*) degrees of freedom (df – column C15); (*iv*) *p*-value (column C16) and (*v*) conclusion (column C17), where two acceptance results are possible: H0 (for null models) or H1 (for alternative models). Through this command, LRTs were performed for all selected M0 and TrU paired rows. To improve figure readability, five columns (from C7 to C11) were hidden, with the command “hide C7-11” (Additional file 2: Figure S7). Remaining aspects of this figure are as explained in Fig. 4 legend, except for the current table indication (“Final Table”) in OMAP PROMPT, here showing the letter “F” (*in red*)

Following the *imap.pl*, the CSV file can be subsequently organized and summarized using the interactive application *omap.pl* (Fig. 4 and Additional file 2: Figure S6), to finally estimate the LRTs and their statistical significance (*p*-value) (Fig. 5). This application comprehends a total of 24 interactive commands (Additional file 2: Figure S7) and two data containers to let the user conveniently manipulate the input data (User Table – Fig. 4 and Final Table – Fig. 5). For the LRTs to be estimated all alternative and null models must be paired in consecutive rows, with the null placed above its alternative model counterpart. Once the statistical confidence value is defined and after issuing “plrt” (e.g.: “plrt 0.05”) (see Additional file 2: Figure S7), five new columns are automatically added to the Final Table, where the estimated results for each test are only displayed in the alternative model rows (Fig. 5).

The *lmap.pl* combines all above described applications in a single action, resembling a computational pipeline (Fig. 1). Proceeding in this manner, the users need only to specify minimal CLOs requirements (Additional file 2: Figure S8), such that no intervention is needed afterwards and until completion, when the researcher is finally required to estimate LRTs. During the *lmap.pl* execution, the CLO -m (Additional file 2: Figure S8) has the advantage to produce in one step all output CSV files from all the models results indicated, as opposed to *imap.pl,* which requires several separate executions. The simplicity of *lmap.pl* is also attained given that it excludes important CLOs (features) that are available in the individual applications, such as the CLO -x from the *mmap.pl* (Additional file 2: Figure S4) or the CLO -t from *gmap.pl* (Additional file 2: Figure S1).

LMAP is a straightforward and useful package to anyone seeking to perform high-throughput analyses of multiple genes or datasets. Through all its applications dismisses the need of manually creating folders and handling (input/output) datasets, editing control files, manual monitoring of *codeml* program executions and retrieval of various ML parameter estimates. Likewise, LMAP allows to automatically organize all results and perform LRTs in endless consecutive pairs of rows using a single interactive command. Additionally, four applications (*cmap.pl*, *mmap.pl*, *imap.pl* and *omap.pl*) are not tied to any special constraints of file identity or formats, rather they can be employed in any existing directory structures that have manually been created by the user. In this way, by adjusting the command-line options accordingly, it is possible to use *cmap.pl* to modify any *codeml* control files as well as to use *mmap.pl* and *imap.pl* to analyze the data and retrieve results, respectively. Furthermore, LMAP generates CSV tables bearing an appropriate format suitable for publication. To conclude, our software solves a variety of difficulties with just a few command-line options and together gives the possibility of receiving an email notification after completion.

Here important advantages standout over the POTION software. Beyond the incorporation of the BRM and phylogenetic tree labeling functionality, LMAP enables additional executions to avoid local optima and provides improved installation procedures (see Availability and requirements section). Additionally, LMAP makes the terminal more appealing to users, by providing a more structured and informative visualization further enhanced by the use of colors (Figs. 2, 3, 4 and 5).

Presently, LMAP has been developed to perform the analyses with the codon substitution models from the *codeml* program from the PAML package. Further features to make LMAP even more versatile will be incorporated in the near future. Nonetheless, LMAP is not applicable in Windows OS due to its main dependency on the *screen* utility program. This required compatibility feature, could be solved through the development of a Graphical User Interface (GUI). It would be interesting to develop LMAP further, by incorporating other kinds of *codeml* analyses, such as amino acid substitution models, and include other PAML programs, such as *baseml* or others. Regardless, the LMAP package will be continuously improved and updated towards the researcher’s needs, which has been accomplished by its application in several ongoing research studies in our group.

### Example dataset and benchmarking

An example dataset is provided in LMAP archive to help users explore and experience the workflow of the package. This folder (“ExampleDataset”) contains two directories, one for MSAs and the other for phylogenetic trees, with files properly identified. A total of 20 whole mitochondrial genome sequences from turtle species (9 freshwater from the superfamily Tryonichia—Tryonichidae and Carettochelyidae—and 11 terrestrial from the family Testudinidae) were retrieved from the online NCBI database. From this survey, 12 mitochondrial DNA (mtDNA) protein-coding genes (ATP6, ATP8, COX1, COX2, COX3, CYTB, ND1, ND2, ND3, ND4, ND4L, ND5) were selected, which additionally compose the concatenated alignment, designated as TMConc2.

To execute LMAP, simply type the following in the command-line: “lmap.pl -A ./ExampleDataset/msas/ -T ./ExampleDataset/trees/ -d . -j ExampleDatasetResults -m s[0:1:2:3:7:8:8a],b,c,w -n 32 --no-omap”. Through CLOs -A and -T, the input files are retrieved to subsequently run the selected models (CLO -m). Likewise, the CLO -j identifies the main directory structure (“ExampleDatasetResults“), which will contain all results (see Additional file 3: Tables S1–S4) and is generated in the folder specified by CLO -d “.” (current directory). To fulfil the workstation CPU capacity, the maximum number of desired tasks was indicated through the CLO -n, which in our example was 32. For purposes of benchmarking, through the inclusion of the CLO --no-omap, the idle execution time from *omap.pl* was avoided forcing *imap.pl* to be executed last.

The output of this command-line originated 2690 *codeml* instances that took 11 h, 55 min and 43 s to complete. This was measured in the UNIX *time* [51] utility program, by using a single workstation configured with 64 GB of RAM and two Intel Xeon E5-2650v2 processors, which together yield a total of 32 hyper-threading cores. In contrast, using a single core, the same instances would take about 322 h, 20 min and 18 s (13 days). To summarize, our package does not interfere in the execution time required by PAML, but instead mitigates how much the researcher spends overseeing each step of the workflow, from the moment the input files are ready to be analyzed, which may be none or minimal.

## Conclusions

We have developed a simple, versatile and highly customizable package named, Lightweight Multigene/Multi-core Analyses in PAML (LMAP) that readily enables the employment of different *codeml* models of molecular adaptive evolution (SM, BM, BSM and CM) and makes possible the analyses of a large number of datasets. At minimum, two files with the appropriate identity are required within a single input directory: one for the MSA and the other for the phylogenetic tree. From this instant, LMAP automatically creates, organizes, executes and extracts all information from the *codeml* results. Thereon, the user is required to manipulate and organize (sorting, selecting, moving, etc.) possibly hundreds or thousands of rows (models results) of his/her dataset in order to accomplish the LRT estimations. Despite this mediation, the process is much simpler than if performed with often slow spreadsheets. Additionally, LMAP allows users to carry out phylogenetic tree labeling; as well as to monitor and control executing *codeml* tasks; re-run datasets which might not have correctly finished and last but not least, receive an email notification when results are ready. To our knowledge, currently there is no other software that combines in one all the described *codeml* models. LMAP has been developed as an open-source command-line and interactive package of tools, allowing its integration into more complex open-source bioinformatics pipelines.

## Availability and requirements

Project Name: LMAP

Project Home Page: http://lmapaml.sourceforge.net/

Operating System: Linux/UNIX and MacOS

Programming Language: Perl

Other Requirements: *codeml* (PAML package version (minimum) 4.6), CPAN modules (IO::All, Email::MIME, Email::Sender, Sys::Info, Term::Readkey, Thread::Semaphore, Statistics::Distributions, Math::Cephes, Bio::TreeIO, File::Copy, File::Copy::Recursive), *screen* and *sendmail* UNIX command-line utilities.

License: GNU General Public License, version 3.0 (GPLv3)

Any restrictions to use by non-academics: no restrictions except the ones stated in GPLv3.

### Installation

The LMAP package provides two additional applications to easily enable LMAP functionality and installation: *(i)* the *install.pl* to enable the installation of all CPAN modules and utilities and *(ii)* the *configure.pl* to enable the configuration of LMAP package. A manual with detailed instructions is included in the archive to allow LMAP user-friendly installation and application.

## Additional files

Additional file 1: Tables presenting templates definition and summary of models comparison. **Table S1.** SM templates defined by the *codeml* control file parameters values and summary of LRT comparisons. **Table S2.** BM templates defined by the *codeml* control file parameters values and summary of LRT comparisons. **Table S3.** BSM templates defined by the *codeml* control file parameters values and summary of LRT comparisons. **Table S4.** CM templates defined by the *codeml* control file parameters values and summary of LRT comparisons. (PDF 278 kb) Additional file 2: Figures exhibiting LMAP applications options. **Figure S1.** command-line options for *gmap.pl* application. **Figure S2.** interactive commands for *gmap.pl* application. **Figure S3.** command-line options for *cmap.pl* application. **Figure S4.** command-line options for *mmap.pl* application. **Figure S5.** command-line options for *imap.pl* application. **Figure S6.** command-line options for the *omap.pl* application. **Figure S7.** interactive commands for *omap.pl* application. **Figure S8.** command-line options for the main *lmap.pl* application. (PDF 4641 kb) Additional file 3: Resulting tables from LMAP execution of the included mitochondrial protein-coding genes dataset. **Table S1.** *imap.pl* results file from BM. **Table S2.** *imap.pl* results file from BSM. **Table S3.** *imap.pl* results file from CM. **Table S4.** *imap.pl* results file from SM. (XLSX 247 kb)

## Abbreviations

BEB: Bayes empirical bayes
BM: Branch models
BRM: Branch related models (BM, BSM and CM)
BSM: Branch-site models
CLO: Command-line option
CM: Clade models
CmC: Clade model C
CPAN: Comprehensive Perl archive network
CPU: Central processing unit
CSV: Comma-separated values
*d*_N_: Number of non-synonymous substitutions
*d*_S_: Number of synonymous substitutions
GNU: GNU’s Not Unix!
GPLv3: GNU general public license version 3.0
GRID: Global resource and information database
GUI: Graphical user interface
HR: Hypothesis reference
IDEA: Interactive display for evolutionary analyses
IMPACT_S: Integrated multiprogram platform to analyze and combine tests of selection
JCoDa: Java codon delimited alignment
LMAP: Lightweight multigene analyses in PAML
lnL: log-Likelihood
LRT: Likelihood ratio test
M0: Model 0
M1a: Model 1a
M2a: Model 2a
M2a_rel: Model 2a_rel
M3: Model 3
M7: Model 7
M8: Model 8
M8a: Model 8a
MA: Model alternative
MA1: Model alternative (with ω = 1)
ML: Maximum likelihood
MSA: Multiple sequence alignment
NCBI: National Center for Biotechnology Information
OS: Operating system
PAML: Phylogenetic analysis by maximum likelihood
Perl: Practical extraction and report language
POTION: POsitive selecTION
PSP: Prokaryotic selection pressure
SM: Site models
TrC: Two-ratio constrained model (with ω = 1)
TrU: Two-ratio unconstrained model
VESPA: Very large-scale evolutionary and selective pressure analyses
