# Supplementary material for: LMAP: Lightweight Multigene Analyses in PAML
Source: BMC Bioinformatics. 2016 Sep 6;17(1):354. doi: 10.1186/s12859-016-1204-5 (PMC5011788; doi:10.1186/s12859-016-1204-5)
Supplement: Additional file 1: — Tables presenting templates definition and summary of models comparison. Table S1. SM templates defined by the codeml control file parameters values and summary of LRT comparisons. Table S2. BM templates defined by the codeml control file parameters values and summary of LRT comparisons. Table S3. BSM templates defined by the codeml control file parameters values and summary of LRT comparisons. Table S4. CM templates defined by the codeml control file parameters values and summary of LRT comparisons. (PDF 278 kb) [file 12859_2016_1204_MOESM1_ESM.pdf]

Table S1: SM templates defined by the *codeml* control file parameters values and summary of LRT comparisons.

**PAML Parameters**	**Site Model**						
	**M0**	**M3**	**M1a**	**M2a**	**M7**	**M8**	**M8a**
seqfile (*)	***automatic***						
treefile (*)	***automatic***						
outfile	R						
noisy	9						
verbose	1						
runmode	0						
seqtype	1						
CodonFreq	2 (F3x4)						
clock	0						
aaDist	0						
model	0						
Nssites (*)	***0***	***3***	***1***	***2***	***7***	***8***	8
icode (*)	***automatic***						
Mgene	0						
fix_kappa	0 (estimate)						
kappa (*)	***0.2 ; 2 ; 5***						
fix_omega	0 (estimate)						1 (fixed)
omega	0.4						1
fix_alpha	1						
alpha	0						
Malpha	0						
ncatG	(**)						4
getSE	0						
RateAncestor	0						
Small_Diff	0.5e-6						
cleandata	1						
fix_blength	1						
method	0						
**Positive selection**	None	Variation of ω among codons	None	Allows positively selected sites	None	Allows positively selected sites	False positive (relaxed purifying selection)
**np**	1	5	2	4	2	4	3
**LRT**	M0 vs. M3		M1a vs. M2a		M7 vs. M8		
						M8a vs. M8	
**df = np(H1) - np(H0)**	4		2		2		
						1	
**BEB**	No	No	No	Yes	No	Yes	No

NOTE: (*) These values are automatically changed by LMAP applications and must not be manually modified by users. Remaining values can and should be modified in respect to their requirements. (**) Any values for this parameter are ignored when running site models in batch mode, whereas *codeml* employs default values. **Legend**: np – number of parameters; LRT – Likelihood Ratio Test; H0 – Null Hypothesis; H1 – Alternative Hypothesis; df – degrees of freedom; BEB – Bayes Empirical Bayes.

Table S2: BM templates defined by the *codeml* control file parameters values and summary of LRT comparisons.

<table>
<tr><th rowspan="2">PAML Parameters</th><th colspan="3">Branch Model</th></tr>
<tr><th>M0</th><th>TrU</th><th>TrC</th></tr>
<tr><td>seqfile (*)</td><td colspan="3">automatic</td></tr>
<tr><td>treefile (*)</td><td colspan="3">automatic</td></tr>
<tr><td>outfile</td><td colspan="3">R</td></tr>
<tr><td>noisy</td><td colspan="3">9</td></tr>
<tr><td>verbose</td><td colspan="3">1</td></tr>
<tr><td>runmode</td><td colspan="3">0</td></tr>
<tr><td>seqtype</td><td colspan="3">1</td></tr>
<tr><td>CodonFreq</td><td colspan="3">2 (F3x4)</td></tr>
<tr><td>clock</td><td colspan="3">0</td></tr>
<tr><td>aaDist</td><td colspan="3">0</td></tr>
<tr><td>model</td><td>0</td><td colspan="2">2</td></tr>
<tr><td>Nssites</td><td colspan="3">0</td></tr>
<tr><td>icode (*)</td><td colspan="3">automatic</td></tr>
<tr><td>Mgene</td><td colspan="3">0</td></tr>
<tr><td>fix_kappa</td><td colspan="3">0 (estimate)</td></tr>
<tr><td>kappa</td><td colspan="3">2</td></tr>
<tr><td>fix_omega</td><td colspan="2">0 (estimate)</td><td>1 (fixed)</td></tr>
<tr><td>omega (*)</td><td colspan="2">0.0 ; 0.1 ; 0.01 ; 0.001 ; 0.25 ; 0.5 ; 0.75 ; 1 ; 1.5 ; 2</td><td>1</td></tr>
<tr><td>fix_alpha</td><td colspan="3">1</td></tr>
<tr><td>alpha</td><td colspan="3">0</td></tr>
<tr><td>Malpha</td><td colspan="3">0</td></tr>
<tr><td>ncatG</td><td>2</td><td colspan="2">4</td></tr>
<tr><td>getSE</td><td colspan="3">0</td></tr>
<tr><td>RateAncestor</td><td colspan="3">0</td></tr>
<tr><td>Small_Diff</td><td colspan="3">0.5e-6</td></tr>
<tr><td>cleandata</td><td colspan="3">1</td></tr>
<tr><td>fix_blength</td><td colspan="3">1</td></tr>
<tr><td>method</td><td colspan="3">0</td></tr>
<tr><td>Divergence</td><td>None (ωF = ωB)</td><td>Divergence (ωF > 1)</td><td>Relaxed selection
(ωF = 1)</td></tr>
<tr><td>np</td><td>1</td><td>2</td><td>1</td></tr>
<tr><td rowspan="2">LRT</td><td colspan="2">M0 vs. TrU</td><td></td></tr>
<tr><td></td><td colspan="2">TrC vs. TrU</td></tr>
<tr><td rowspan="2">df = np(H1) - np(H0)</td><td colspan="2">1</td><td></td></tr>
<tr><td></td><td colspan="2">1</td></tr>
<tr><td>BEB</td><td>No</td><td>No</td><td>No</td></tr>
</table>

NOTE: (*) These values are automatically changed by LMAP applications and must not be manually modified by users. Remaining values can and should be modified in respect to their requirements. **Legend**: ωF – Foreground omega; ωB – Background omega; np – number of parameters; LRT – Likelihood Ratio Test; H0 – Null Hypothesis; H1 – Alternative Hypothesis; df – degrees of freedom; BEB – Bayes Empirical Bayes.

Table S3: BSM templates defined by the *codeml* control file parameters values and summary of LRT comparisons.

PAML Parameters	Branch-Site Model	
	MA1	MA
seqfile (*)	***automatic***	
treefile (*)	***automatic***	
outfile	R	
noisy	9	
verbose	1	
runmode	0	
seqtype	1	
CodonFreq	2 (F3x4)	
clock	0	
aaDist	0	
model	2	
Nssites	2	
icode (*)	***automatic***	
Mgene	0	
fix_kappa	0 (estimate)	
kappa (*)	***0.2 ; 2 ; 5***	
fix_omega	1 (fixed)	0 (estimate)
omega	1	1.5
fix_alpha	1	
alpha	0	
Malpha	0	
ncatG	(**)	
getSE	0	
RateAncestor	0	
Small_Diff	0.5e-6	
cleandata	1	
fix_blength	1	
Method	0	
**Positive selection**	None (ωF = ωB)	Allows positive selected sites (ωF > 1)
**np**	1	2
**LRT**	MA1 vs. MA	
**df = np(H1) - np(H0)**	1	
**BEB**	No	Yes

NOTE: (*) These values are automatically changed by LMAP applications and must not be manually modified by users. Remaining values can and should be modified in respect to their requirements. (**) Any value in this parameter is ignored in both branch-site models A e B, whereas *codeml* employs default values. **Legend**: ωF – Foreground omega; ωB – Background omega; np – number of parameters; LRT – Likelihood Ratio Test; H0 – Null Hypothesis; H1 – Alternative Hypothesis; df – degrees of freedom; BEB – Bayes Empirical Bayes.

Table S4: CM templates defined by the *codeml* control file parameters values and summary of LRT comparisons.

PAML Parameters	Clade Model	
	M2a_rel	CmC
seqfile (*)	***user***	
treefile (*)	***user***	
outfile	R	
noisy	9	
verbose	1	
runmode	0	
seqtype	1	
CodonFreq	2 (F3x4)	
clock	0	
aaDist	0	
model	0	3
Nssites	22	2
icode (*)	***user***	
Mgene	0	
fix_kappa	0 (estimate)	
kappa	2	
fix_omega	0 (estimate)	
omega (*)	***0.0 ; 0.1 ; 0.01 ; 0.001 ; 0.25 ; 0.5 ; 0.75 ; 1 ; 1.5 ; 2***	
fix_alpha	1	
alpha	0	
Malpha	0	
ncatG	(**)	
getSE	0	
RateAncestor	0	
Small_Diff	0.5e-6	
cleandata	1	
fix_blength	1	
method	0	
**Divergence**	None (ωF = ωB)	Divergence (ωF > 1)
**np**	1	2
**LRT**	M2a_rel vs. CmC	
**df = np(H1) - np(H0)**	1	
**BEB**	No	Yes

NOTE: (*) These parameters are automatically changed by LMAP applications and must not be manually modified by users. Remaining parameters can and should be modified in respect to their requirements. (**) Any value in this parameter is ignored in both clade model C and M2a_rel models, whereas *codeml* employs default values. **Legend**: ωF – Foreground omega; ωB – Background omega; np – number of parameters; LRT – Likelihood Ratio Test; H0 – Null Hypothesis; H1 – Alternative Hypothesis; df – degrees of freedom; BEB – Bayes Empirical Bayes.
