# Supplementary material for: LMAP: Lightweight Multigene Analyses in PAML
Source: BMC Bioinformatics. 2016 Sep 6;17(1):354. doi: 10.1186/s12859-016-1204-5 (PMC5011788; doi:10.1186/s12859-016-1204-5)
Supplement: Additional file 2: — Figures exhibiting LMAP applications options. Figure S1. command-line options for gmap.pl application. Figure S2. interactive commands for gmap.pl application. Figure S3. command-line options for cmap.pl application. Figure S4. command-line options for mmap.pl application. Figure S5. command-line options for imap.pl application. Figure S6. command-line options for the omap.pl application. Figure S7. interactive commands for omap.pl application. Figure S8. command-line options for the main lmap.pl application. (PDF 4641 kb) [file 12859_2016_1204_MOESM2_ESM.pdf]

Figure S1: command-line options for *gmap.pl* application.

```
NAME:

  gmap.pl - Generate an organized directory structure.

SYNOPSIS:

  gmap.pl -A [dirfiles] -T [dirfiles] -d [location] -m [s[models],b,w,c] {-j [projname]} {-O b[x,y,...,z]} {-K s[x,y,...,z]}
  gmap.pl -A [dirfiles] -t [inittree.nwk] -d [location] -m [s[models],b,w,c] {-j [projname]} {-O c[x,y,...,z]} {-K w[x,y,...,z]}

DESCRIPTION:
  |  -------------------------------------------------------------------------
  | This application is intended to generate an organized directory structure, containing all
  | necessary files for the execution of codeml from PAML package. All files, alignments and phylogenetic trees,
  | can be supplied by the user in separate directories. These files need to be named specially to the
  | purpose of distributing files to the correct locations in the final directory. Final control files are obtained
  | through the templates supplied with the package, which are automatically modified according to the options selected by
  | the user and placed along with the respective alignment and phylogenetic tree.
  | A specific option allows the user to make several interactive edits in an initial rooted phylogenetic tree.
  | Among the possible editions, the most relevant is the placement of PAML specific signs for branch (#) and clade ($),
  | thus enabling the labeling of phylogenetic trees, required for branch, branch-site and clade models.
  | An interactive help is provided, to help the user to perform these and other modifications.
  | The resulting directory structure can then be passed into mmap.pl application for running.

OPTIONS:
  |  -------------------------------------------------------------------------
  | -A [algndir]    Input directory containing all the alignment files distinguished by its name.
  |                 The name for each alignment file must contain gene name abbreviation (eg.: ND5)
  |                 followed by the models indication (see option -m below) for which this alignment will be used and followed by
  |                 one or two digits indicating the PAML icode parameter for each alignment. Gene name must be separated by '_'
  |                 (underscore) from the rest. Formats supported: FASTA (.fst, .fas, .fasta) and PHYLIP (.ph, .phy, .phylip).
  |                    eg.: [algndir]/ND5_sbc0.fas ; with icode 0 (universal code) will be employed in site, branch and clade models.
  | -T [treedir]    Input directory containing all the phylogenetic tree files (in NEWICK format) to be applied in several model types
  |                 (see option -m below). These are distinguished by its name, which must be given as the name of each hypothesis to
  |                 test or otherwise, for the only case of site-models, simply the matching gene names.
  |                    eg.: [treedir]/H_bc.nwk ; the hypothesis is named as 'H' and this tree is to be employed in branch and
  |                           clade models ('b' and 'c' options are according to option -m arguments found below)
  |                    eg.: [treedir]/ND5_s.nwk ; this tree is to be used for the case of site models for ND5 gene/alignment (in option -A).
  | -t [tree.nwk]   Input single tree file, in NEWICK format, for labeling in interactive mode. Use instead of the option -T.
  |                 It enables the user to edit/label and save phylogenetic trees for multiple hypothesis testing, for branch,
  |                 branch-site and clade models. PAML tags can be inserted for branches (#) or clades ($).
  | -d [dir]        Input directory which will contain the final directory structure containing all input files.
  | -m [typmodels]  One or more of the following options in any order, separated by commas. eg.: -m c,s[0:1:2:3:7:8:8a],b,w.
  |    s[a:...:z]      Prepare to run the specified site models. Where a:...:z are any values/models from (0,1,2,3,7,8,8a),
  |                     separated by ':' and enclosed in brackets '[]'.
  |    b               Prepare to run branch models. Will run models M0, TrC and TrU.
  |    w               Prepare to run branch-site models. Will run models A (Alternative) and A1 (Null).
  |    c               Prepare to run clade models. Will run models CmC (Alternative) and M2a_rel (Null).
  |                 ----------------------- not mandatory: ----------------------
  | -j [projname]   Specify the project name. This is part of the directory structure to be created and will be
  |                 the base directory name. Hereafter, it can be used to build additional runs for other
  |                 model types at this location. By default, a generic name will be created and used.
  | -O [b[o]:c[o]]  Define the omega values to be tested for each model in option -m. By default, the omega values used in
  |                 the case of branch and clade models are: 0.0, 0.001, 0.01, 0.1, 0.25, 0.5, 0.75, 1, 1.5, 2
  |                 The values can be specified for one or both model types (b or c), in any order. To specify which values
  |                 to run for each model, type as in following examples: -O b[x,y,...,z]:c[x,y,...,z] or -O c[x,y,...,z]
  | -K [s[k]:w[k]]  Define the kappa values to be tested for each model in option -m. By default, the kappa values used in
  |                 the case of branch-site and site models are: 0.2, 2, 5
  |                 The values can be specified for one or both model types (s or w), in any order. To specify which values
  |                 to run for each model, type as in following examples: -K s[x,y,...,z]:w[x,y,...,z] or -K w[x,y,...,z]
  |                 -----------------------------------
  | --help          Dump the interactive commands help to a file located in current directory, named 'gmap_promptHelp.txt'.
  | -h              This help
  | -v              Application version

VERSION: 1.0.0 Nov 20th, 2015
```

```
        GMAP PROMPT HELP - COMMANDS
================================================================================

Options in [ ] - means, options are not mandatory; | - means, alternative options; ( ) - means, grouping

NOTE: The BioPerl modules here used, require that the inicial/input tree is rooted.
See http://www.bioperl.org/wiki/HOWTO:Trees

Command    Options         Description
========== =============== ======================
rbl                        Remove or clear branch length values, when available on the tree.
rbs                        Remove or clear bootstrap values, when available on the tree.
(Lx|Lx,...,Lz)=(#)y        Specify one or more species nodes (leafs) to be labeled with PAML symbols for branch (#),
                           followed by any required number eg.: L11=#1 or L07,L09,L11=#1.
(Ax|Ax,...,Az)=($|#)y      Specify one or more ancestral nodes to be labeled with PAML symbols for branch (#) or for clade($),
                           followed by any required number eg.: A11=#1 or A07,A09,A11=$1.
ls         [path|dir]      List contents on given directory. If no argument is given, lists current directory.
save       filename.nwk    Save tree with current configuration to filename.nwk. Default directory is ./lmap_savedtrees
saveunr    filename.nwk    Save rooted tree as unrooted with current configuration to filename.nwk. Default directory is ./lmap_savedtrees
savetodir  path|dir        Set default directory where to save (labeled) newick trees.
                           Will change default directory ./lmap_savedtrees to the one given as argument.
show       bs|bl|b|n       Show values/labels on the tree. Options are bs: bootstraps ; bl: branch-lengths ; b: both ; n:none.
brlen      n               Set branch length for display, n number of characters. By default is set to 100.
rootunr    Na,Nb           Root an unrooted tree, by specifying two nodes. This command creates a new node having Na and Nb as descendents
                           and the current 'root' as ancestor. This new node will hence be located below the current 'root' and above the specified descendents.
                           Nodes Na and Nb are any two nodes, leafs or ancestrals, connected to the current 'root'. eg.: rootunr A16,L01
                           Note: the word root was employed in quotes ('root'), since the node presented as most close to the left is not a true root.
                           In fact, by BioPerl design, every tree must be rooted and every unrooted tree has thus an additional root node,
                           which in this case becomes evident. Hence, the root displayed is in fact, a false root.
prune      (Nx|Nx,...,Nz)  Prune one or more nodes specified from phylogenetic tree, without changing the tree relationships.
                           Nodes can be tips (leafs) or ancestrals, in which case, the entire clade or descendent branches are removed,
                           including the specified ancestral node. eg.: prune A13 (ancestral) or prune L11 (leaf) or prune L11,L12,A17 (leafs and ancestral).
rtaxa      (Lx=N,...,Lz=N) Rename phylogenetic tree taxa. This can be done with two alternative options: either by supplying an option
           |(file.csv)     consisting of 1 or more pairs of node/taxa (Lnumber) and associated new name (N), separated by commas, as in
                           (L01=Name1,L02=Name2,...); or an option indicating any titled CSV file listing two columns, containing at
                           the left column, the original name and at the right column, the new name.
                           eg.: rtaxa L11=Ted or rtaxa L11=Ed,L78=Tom,L80=Joe or rtaxa path/to/filename.csv.
reset                      Reread tree from file, thus forgetting any previous changes made.
---------- --------------- ----------------------
d[one]                     Verifies if tree was saved, before exiting.
?                          This Help.
========== =============== ======================

VERSION: 1.0.0 Nov 20th, 2015
================================================================================
```

Figure S2: interactive commands for *gmap.pl* application.

```
NAME:

  cmap.pl - Change PAML control files specific parameters.

SYNOPSIS:

  cmap.pl -d [directory] -f [e:ctl] -g f:param -L "value" {-r}

DESCRIPTION:
  |  -------------------------------------------------------------------------
  | This application is intended to change specific parameters values in several
  | PAML control files already located in an organized directory structure.
  | This is a helper application which is meant to be used, if necessary, after
  | creating an organized directory structure, done by gmap.pl and before running
  | with mmap.pl, so that, any required changes can still take effect.

OPTIONS:
  |  -------------------------------------------------------------------------
  | -d [dir]         Input directory of the final structure containing all PAML input files.
  | -f [tag:part]    Choose control files through a given filename portion, where tag is one of:
  |                      f: filename equals given part
  |                      s: filename starts with part
  |                      e: filename ends with part
  | -g [tag:ptext]   Control file target parameter line identified by its name eg.: -g f:icode
  |                  Where tag is one of the following:
  |                      f: Complete/full line
  |                      s: Starting with text  (may affect several lines/parameters)
  |                      c: Containing text     (may affect several lines/parameters)
  |                      e: Ending with text    (may affect several lines/parameters)
  | -L [paramval]    Value or text to be replaced in selected line(s)/parameter(s) from option -g.
  |                  ----------------------- not mandatory: ---------------------
  | -r               Work recursively.
  |                  -----------------------------------
  | -h               This help
  | -v               Application version

VERSION: 1.0.0 Nov 20th, 2015
```

Figure S3: command-line options for *cmap.pl* application.

```
NAME:

  mmap.pl - Run multiple PAML instances.

SYNOPSIS:

  mmap.pl -d [dirpath] {-r} {-n [int]} {-f [e:ctl]} {-p [l:progname]} {-R [resfilename]} {-l} {-e {emailaddr}}

DESCRIPTION:
  |  ---------------------------------------------------------------------------
  | This application is intended to run and monitor multiple PAML (codeml) instances,
  | from an organized directory struture, containing all necessary data files.
  | This organized directory structure can be previously created by using the gmap.pl application.
  | The results from this/these runs can then be collected with the application imap.pl.

OPTIONS:
  |  ---------------------------------------------------------------------------
  | -d [dir]        Input directory containing all necessary files.
  |                 ----------------------- not mandatory: ----------------------
  | -R [filename]   Results filename common to all PAML runs. By default, is the same occuring in templates files: R
  | -f [tag:part]   Choose control files through a given filename portion, where tag is one of:
  |                      f: filename equals given part
  |                      s: filename starts with part
  |                      e: filename ends with part
  |                 By default, is e:ctl based on the common codeml control filename: codeml.ctl
  | -p [tag:prog]   Program to run, where tag is one of:
  |                      l: from default location eg.: l:progname
  |                      f: given full path eg.: f:path/to/progname
  |                 For tag 'l' the location defaults to HOME/bin/ or the one chosen by the user during installation.
  |                 Use tag 'f' to give a different location for the intended PAML executable(s).
  |                 By default, is l:codeml based on the default PAML executables location : /home/labpc3c/bin
  | -r              Work recursively.
  | -x              Use this option to rerun a previous directory, which may have files
  |                 not completed or PAML runs previously not initiated.
  | -n [integer]    Specify the maximum number of CPUs/cores available (limit) to use for the current task.
  |                 By default will try to use the aproximate maximum number of cores available.
  |                 This option can be usefull to enable sharing of the CPU capacity between users or different (mmap) tasks.
  | -l              Enable logging of all the finished runs in a CSV file located at the current working directory.
  | -e {emailaddr}  Enable email notification, when mmap.pl finishes. The argument to this option, is optional and has two meanings:
  |                 If email address is not specified, it will default to the address defined during installation. Otherwise, it
  |                 will be given preference to the address here supplied. In case the address is not given in either case,
  |                 (during installation and in this option), notification will not be sent.
  |                 -----------------------------------
  | -h              This help
  | -v              Application version

VERSION: 1.0.0 Nov 20th, 2015
```

Figure S4: command-line options for *mmap.pl* application.

```
NAME:

  imap.pl - Extract PAML information from results files.

SYNOPSIS:

  imap.pl -d [dirstruct] [-s [models] | -b | -w | -c] -o [outfile.csv] {-R [res.filename]} {-r}

DESCRIPTION:
  |  ---------------------------------------------------------------------------
  | This application is intended to extract PAML (codeml) information from the results files located
  | in an organized directory struture, which contain data regarding any models previously run.
  | The information extracted is conveniently placed in CSV tables consisting of several important parameters/values.
  | The resulting file can then be passed as input into omap.pl, for screening and final LRT calculations.

OPTIONS:
  |  ---------------------------------------------------------------------------
  | -d [dir]        Input directory containing all and only the directories that contain the codeml files.
  | -s [0,...,n]    Extract information from the specified site models results.
  | -b              Extract information from branch models results.
  | -w              Extract information from branch-site models results.
  | -c              Extract information from clade models results.
  | -o [outfile]    Choose a name for the resulting CSV file to create or append results.
  |                 ----------------------- not mandatory: ---------------------
  | -r              Work recursively.
  | -R [filename]   Results filename common to all PAML runs. By default, is the same occurring in templates files: R
  |                 ----------------------------------
  | -h              This help
  | -v              Application version

VERSION: 1.0.0 Nov 20th, 2015
```

Figure S5: command-line options for *imap.pl* application.

```
NAME:

  omap.pl - Organize CSV files from imap.pl and perform LRT tests.

SYNOPSIS:

  omap.pl -i [infile.csv] -o [outfile.csv]

DESCRIPTION:
  |  ---------------------------------------------------------------------------
  | This application is intended to interactively organize PAML (codeml) results extracted and
  | collected into a CSV file with the application imap.pl. These results can be organized
  | through several steps required and performed by the user. omap.pl provides several
  | interactive commands that help the user easily achieve this purpose and to finally
  | perform the necessary LRT tests.
  | Information about the interactive commands is provided interactively, by typing '?'.

OPTIONS:
  |  ---------------------------------------------------------------------------
  | -i [infile]     CSV results file from imap.pl.
  | -o [outfile]    Choose a name for the resulting CSV file to create or append results.
  |                 -----------------------------------
  | --help          Dump the interactive commands help to a file located in current directory, named 'omap_promptHelp.txt'.
  | -h              This help
  | -v              Application version

VERSION: 1.0.0 Nov 20th, 2015
```

Figure S6: command-line options for the *omap.pl* application.

```
        OMAP PROMPT HELP - COMMANDS
================================================================================

Options in [ ] - means, options are not mandatory; | - means, alternative options; ( ) - means, grouping

Command    Options         Description
========== =============== ======================
open       filename.csv    (Only in User Table! see command show) Open CSV file, discarding any current values.
ls         [path|dir]      List contents on given directory. If no option is given, lists current directory.
reset                      Reset contents in User Table to initial contents.
delcl(*)   C(a|a-z|a,d,h)  Remove column(s). Either a single column or a range of columns specifed by from-to values or any columns comma-separated.
hide       C(a|a-z|a,d,h)  (Un)Hide column(s). Either a single column or a range of columns specifed by from-to values or any columns comma-separated.
sort(*)    Cn (A|a)|(D|d)  Sort column C[number] [A|a]scending or [D|d]escending. This is performed according to its contents type.
show(*)    (n|t)|(n t)     Show i) (number of rows n) or (Table t) or ii) number of rows (n) in Table (t). (t = [U|u]ser or [F|f]inal).
mark(*)    [a|a-z|a,d,h]   (Un)Mark rows. Either a single row, a range of rows specifed by from-to values or any rows comma-separated;
                           If no option is given, the selection will (un)mark all rows in Table.
markf(*)   r Cn [l]        (Un)Mark the first r rows which contain distinct values in column C[number];
                           Search within l rows limit or, by default, in full Table length.
findr(*)   "value" Cn      (Un)Mark all rows containing the quoted specified value in column C[number];
                           The characters '^','$','|','[' and ']', can be used in the value to mean either 'start', 'end', 'or', or 'set of alternative characters', respectively;
                           eg.: findr "^value" C4 ; findr "value$" C4 ; findr "val1|val2|val3" C4 ; findr "[vV]alue" C4.
copy(*)    [n]             (Only in User Table! see command show) Copy any marked rows, n times each, from User Table to Final Table. If not given n, number of copies defaults to 1;
                           Copies are differentiated by 'index.subindex' in column 'Index', 2nd from the left. Use command 'findr' to (un)mark any copies.
move(*)                    (Only in User Table! see command show) Move any marked rows from User Table to Final Table.
mrup(*)    [n]             Move any marked rows n positions up in current Table. If not given n, number of positions defaults to 1.
mrdn(*)    [n]             Move any marked rows n positions down in current Table. If not given n, number of positions defaults to 1.
delrs(*)                   Delete any marked rows in current Table.
fh                         Show file headers in current Table.
empty(*)                   Empty Final Table contents.
plrt(*)    significance    (Only in Final Table! see command show) Perform LRT test for every pair of rows, in Final Table, from top to bottom;
                           The null model should be placed on the row above the row of the alternative model. In case rows are marked, this operation is performed preferencially in the selection.
pbeb(*)                    (Only in Final Table! see command show) Create an additional column containing the BEB information considering the LRT test conclusion if exists;
                           otherwise, BEB will appear in all rows regardless of the LRT conclusion. This column can only be placed, if initially existed in the input file.
save[!](*) [file.csv]      (Only in Final Table! see command show) Save Final Table contents to default output file (from initial option -o) or if specified, to 'file.csv';
                           Append exclamation mark at the end of command, as in 'save! file.csv', to save contents and make the specified CSV file the default;
                           The contents will be appended to the end of the default file, whenever the file exists; regardless if the file is empty or is a new file;
                           Only marked rows (see command mark, markf, findr), are saved whenever found in Table; or otherwise, all is saved.
def        [CMD[=LIST]]    Define an alias command to perform several operations at once; two other uses can take place:
                           1) The user chooses a command name (CMD) (not found in this HELP) and a LIST of commands, where LIST=(cmd1;...;cmdn), following previous rules for each command;
                           2) Type 'def CMD' to show the definition for the specified command;
                           3) Type 'def' only to show the list of all the defined commands;
                           (*) The commands allowed in the LIST are here marked by an asterisc.
defsave    [file.name]     Save all defined commands to file. File is by default /home/labpc3c/.omapd, if none is given.
defload    [file.name]     Load all defined commands from file. File is by default /home/labpc3c/.omapd, if none is given.
defdel                     Delete /home/labpc3c/.omapd file.
                           This file may contain several definitions including redefinitions of the same command. Hence, the last definitions in file prevail.
---------- --------------- ----------------------
q[uit]                     Checks if any unsaved data are there in Final Table, before exiting.
?                          This Help.
========== =============== ======================

VERSION: 1.0.0 Nov 20th, 2015
================================================================================
```

Figure S7: interactive commands for *omap.pl* application.

Figure S8: command-line options for the main *lmap.pl* application.

```
NAME:

  lmap.pl - Lightweight Multigene Analyses in PAML.

SYNOPSIS:

  lmap.pl -A [dirfiles] -T [dirfiles] -d [location] -m [s[models],b,w,c] -j [projname]
          {-f [e:ctl]} {-p [l:codeml]} {-R [resfilename]} {-n [integer]} {-e {emailaddr}} {-O [b[x,y,...,z]]} {-K [s[x,y,...,z]]}

DESCRIPTION:
  |  ----------------------------------------------------------------------------
  | This is the main program of LMAP package and is a wrapper of all LMAP applications in the following order:
  |       'gmap.pl > (cmap.pl >) mmap.pl > imap.pl (> omap.pl)'
  |    (Further information can be found in any of the help messages of LMAP applications by using option -h).
  | The purpose is to run all the LMAP applications using only one program. The application omap.pl will always require
  | intervention from the user to proceed with specific operations. At this point omap.pl will be running, ready to be used.
  | The cmap.pl application can be enabled (included in the workflow), by using two extra options (-g and -L).
  | The omap.pl application can be disabled (excluded from the workflow), by specifying the option --no-omap.
  | The use of lmap.pl does not hinder or entirely replace the use of the remaining applications.
  | Please note that many options from the LMAP applications (g/c/m/i/omap.pl) are not available in this context and
  | some have become mandatory in this program, for example, option -j.

OPTIONS:
  |  ----------------------------------------------------------------------------
  | -A [algndir]    Input directory containing all the alignment files distinguished by its name.
  |                 The name for each alignment file must contain gene name abbreviation (eg.: ND5)
  |                 followed by the models indication (see option -m below) in which this alignment will be used and followed by
  |                 one or two digits indicating the PAML icode parameter for each alignment. Gene name must be separated by '_'
  |                 (underscore) from the rest. Formats supported: FASTA (.fst, .fas, .fasta) and PHYLIP (.ph, .phy, .phylip).
  |                    eg.: [algndir]/ND5_sbc0.fas ; with icode 0 (universal code) will be employed in site, branch and clade models.
  | -T [treedir]    Input directory containing all the phylogenetic tree files (in NEWICK format) to be applied in several model types
  |                 (see option -m below). These are distinguished by its name, which must be given as the name of each hypothesis to
  |                 test or otherwise, for the only case of site-models, simply the matching gene names.
  |                    eg.: [treedir]/H_bc.nwk ; the hypothesis is named as 'H' and this tree is to be employed in branch and
  |                         clade models ('b' and 'c' options are according to option -m arguments found below).
  |                    eg.: [treedir]/ND5_s.nwk ; this tree is to be used for the case of site models for ND5 gene/alignment (in option -A).
  | -d [dir]        Input directory which will contain the final directory structure containing all input files.
  | -m [typmodels]  One or more of the following options in any order, separated by commas. eg.: -m c,s[0:1:2:3:7:8:8a],b,w.
  |    s[a:...:z]      Prepare to run the specified site models. Where a:...:z are any values/models from (0,1,2,3,7,8,8a),
  |                     separated by ':' and enclosed in brackets '[]'.
  |    b               Prepare to run branch models. Will run models M0, Trc and Tru.
  |    w               Prepare to run branch-site models. Will run models A (Alternative) and A1 (Null).
  |    c               Prepare to run clade models. Will run models CmC (Alternative) and M2a_rel (Null).
  | -j [projname]   Specify the project name. This is part of the directory structure to be created and will be
  |                 the base directory name. Hereafter, it can be used to build additional runs for other
  |                 model types at this location.
  |                 ----------------------- not mandatory: ---------------------
```

(Continues on next page…)

Figure S8: command-line options for the main *lmap.pl* application. (continued)

```
|                  ---------------------- not mandatory: ---------------------
| -R [filename]    Results filename common to all PAML runs. By default, is the same occuring in templates files: R
| -f [tag:part]    Choose control files through a given filename portion, where tag is one of:
|                       f: filename equals given part
|                       s: filename starts with part
|                       e: filename ends with part
|                  By default, is e:ctl based on the common codeml control filename: codeml.ctl
| -p [tag:prog]    Program to run, where tag is one of:
|                       l: from default location eg.: l:progname
|                       f: given full path eg.: f:path/to/progname
|                  For tag 'l' the location defaults to /home/labpc3c/bin/ or the one chosen by the user during installation.
|                  Use tag 'f' to give a different location for the intended PAML executable(s).
|                  By default, is l:codeml based on the default PAML executables location : /home/labpc3c/bin
| -O [b[o]:c[o]]   Define the omega values to be tested for each model in option -m. By default, the omega values used in
|                  the case of branch and clade models are: 0.0, 0.001, 0.01, 0.1, 0.25, 0.5, 0.75, 1, 1.5, 2
|                  The values can be specified for one or both model types (b or c), in any order. To specify which values
|                  to run for each model, type as in following examples: -O b[x,y,...,z]:c[x,y,...,z] or -O c[x,y,...,z]
| -K [s[k]:w[k]]   Define the kappa values to be tested for each model in option -m. By default, the kappa values used in
|                  the case of branch-site and site models are: 0.2, 2, 5
|                  The values can be specified for one or both model types (s or w), in any order. To specify which values
|                  to run for each model, type as in following examples: -K s[x,y,...,z]:w[x,y,...,z] or -K w[x,y,...,z]
| -n [integer]     Specify the maximum number of CPUs/cores available (limit) to use for the current task.
|                  By default will try to use the aproximate maximum number of cores available.
|                  This option can be usefull to enable sharing of the CPU capacity between users or different (mmap) tasks.
| -e {emailaddr}   Enable email notification, when mmap.pl finishes. The argument to this option, is optional and has two meanings:
|                  If email address is not specified, it will default to the address defined during installation. Otherwise, it
|                  will be given preference to the address here supplied. In case the address is not given in either case
|                  (during installation and in this option), notification will not be sent.
| -g [tag:ptext]   [Enable cmap.pl application (use both -g and -L)].
|                  Control file target parameter line identified by its name eg.: -g f:icode
|                  Where tag is one of the following:
|                      f: Complete/full line
|                      s: Starting with text  (may affect several lines/parameters)
|                      c: Containing text     (may affect several lines/parameters)
|                      e: Ending with text    (may affect several lines/parameters)
| -L [paramval]    [Enable cmap.pl application (use both -g and -L)].
|                  Value or text to be replaced in selected line(s)/parameter(s) from option -g.
| --no-omap        Disable the execution of omap.pl application.
|                  -----------------------------------
| -h               This help
| -v               Script version

VERSION: 1.0.0 Nov 20th, 2015
```
